# Morphometric and genetic differentiation among populations of flat‐headed cusimanse (*Crossarchus platycephalus*) in Nigeria

**DOI:** 10.1002/ece3.4262

**Published:** 2018-06-25

**Authors:** Bukola G. Oguntuase, Babafemi G. Ogunjemite, Richard P. Meisel

**Affiliations:** ^1^ Department of Ecotourism and Wildlife Management Federal University of Technology Akure Nigeria; ^2^ Department of Biology and Biochemistry University of Houston Houston Texas

**Keywords:** conservation, *Crossarchus platycephalus*, population genetics, structure

## Abstract

Geographic barriers can partition genetic diversity among populations and drive evolutionary divergence between populations, promoting the speciation process and affecting conservation goals. We integrated morphological and genomic data to assess the distribution of variation in the flat‐headed cusimanse (*Crossarchus platycephalus*), a species of least conservation concern, on either side of the River Niger in Nigeria. Ecological disturbances affect the conservation status of many other animals in this region. The two populations were differentiated in the snout and fore limbs, with greater morphological diversity in the western population. We used Restriction site Associated DNA sequencing (RAD‐seq) and identified two genotypic clusters in a STRUCTURE analysis. Individuals from the eastern population are almost entirely assigned to one cluster, whereas genotypes from the western population are a mixture of the two clusters. The population from west of the River Niger also had higher heterozygosity. The morphological and population genetic data are therefore in agreement that the population from west of the River Niger is more diverse than the eastern population, and the eastern population contains a subset of the genetic variation found in the western population. Our results demonstrate that combining morphological and genotypic measures of diversity can provide a congruent picture of the distribution of intraspecific variation. The results also suggest that future work should explore the role of the River Niger as a natural barrier to migration in Nigeria.

## 1 INTRODUCTION

The retention of genetic diversity is a critical goal of conservation biologists with interests ranging from preventing the extinction of endangered species to promoting the success of species of least concern (Amos & Balmford, 2001; Avise & Hamrick, 1996). Habitat fragmentation and geographical barriers can limit genetic exchange between populations (Primack, 2002; Templeton, Shaw, Routman, & Davis, 1990). When there is little connectivity or few corridors linking populations, low gene flow between populations and loss of genetic diversity within populations are the expected consequences (Fischer & Lindenmayer, 2007). These conditions can also enhance inbreeding within populations (Templeton et al., 1990).

Advances in genomics now allow assessment of genetic variation within and between populations across many molecular markers without a priori information in nonmodel organisms (Andrews, Good, Miller, Luikart, & Hohenlohe, 2016; Narum, Buerkle, Davey, Miller, & Hohenlohe, 2013). These genomic approaches have also been used to identify associations between genetic variants and phenotypes (*e.g.,* Gagnaire, Normandeau, Pavey, & Bernatchez, 2013; Parchman et al., 2012; Richards et al., 2013; Takahashi, Sota, & Hori, 2013). However, there are at least two concerns about measures of DNA sequence variation in conservation biology. First, there is very little evidence that sequence variation is predictive of current census population sizes (Bazin, Glemin, & Galtier, 2006; Leffler et al., 2012; Lewontin, 1974). Second, the relationship between the amount of sequence variation in natural populations and phenotypic diversity in those same populations is rarely studied (cf. Hoffman et al., 2014; Pissard et al., 2008).

**Figure 1:**
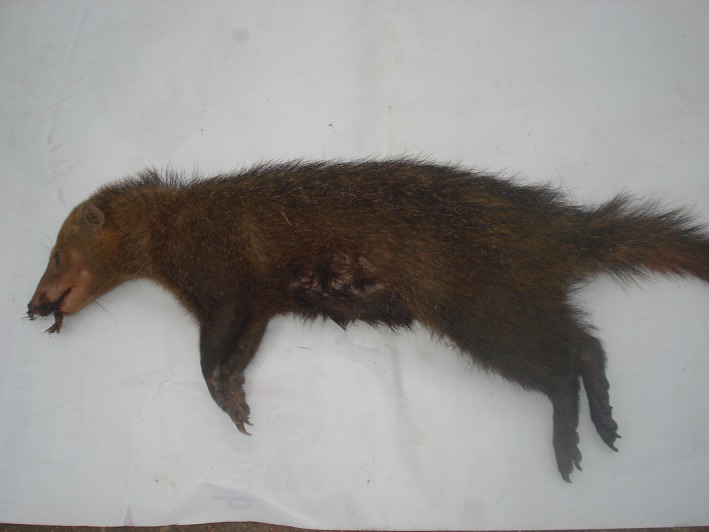
Picture of *Crossarchus platycephalus* individual captured by hunters

We address the relationship between phenotypic and molecular diversity by studying populations of the flat‐headed cusimanse (*Crossarchus platycephalus*) east and west of the River Niger in Nigeria. *C. platycephalus* are small carnivores (Figure 1) belonging to the family Herpestidae, and found in rainforests of Benin, Nigeria, Cameroon, Equatorial Guinea, Congo Republic, and Central African Republic (Wilson & Reeder, 2005). They are social animals with a family composition of mating pairs and their young. In small mongooses, males disperse more frequently than females, and new migrants act as subordinates in the new group they move into (Waser, Elliott, Creel, & Creel, 1995), though they are able to attain alpha rank earlier than the original members of the group (Rood, 1990). Flat‐headed cusimanse eat a variety of food products, ranging from plants to animals (Kingdon, 1997), and the species is considered to be of least concern of extinction (Angelici & Do Linh San, 2016). However, along with some other carnivora hunted in Africa, it is often encountered in the bush meat market (Angelici & Di Vittorio, 2013; Gaubert et al., 2015), suggesting that overhunting could decrease population sizes. Flat‐headed cusimanse is therefore a good model to study the preservation of genetic diversity prior to population decline.

The range of *C. platycephalus* in Nigeria is divided by the major rivers Niger and Benue. Along with other natural barriers, rivers restrict the distributions of many African mammals (Anthony et al., 2007; Booth, 1958; Clifford et al., 2004; Groves, 2001; Grubb et al., 2003; Kingdon, 1997; Moreau, 1969; Oates, Bergl, & Linder, 2004; Robbins, 1978). For example, the Sanaga River contributes to genetic differentiation between chimpanzee populations across Cameroon and eastern Nigeria (Matthew, Sabrina, Paul, Henri, & Mary, 2015). In addition, Nigeria's population is growing, and people continue to encroach into and further fragment ecologically important areas for agricultural purposes (CIA, 2008). These activities could affect the habitats of wild animal species, which might have consequence on gene flow between populations with conservation implications.

To address the relationship between genotypic and phenotypic variation in potentially subdivided populations, we assessed DNA sequence and morphological variation in *C. platycephalus* on either side of the River Niger. There are no previous studies of population size in *C. platycephalus* and no documented studies on the structure and genetic diversity of the populations in Nigeria. There are also no diagnostic differences in physical appearance to distinguish between the eastern and western populations, although flat‐headed cusimanse can be distinguished from their sister species, *Crossarchus obscurus*, based on skull morphology (Goldman, 1984; Kingdon et al., 2013; Wilson & Reeder, 2005) and genetic markers (Olayemi et al., 2011; Sonet et al., 2014; Veron, Colyn, Dunham, Taylor, & Gaubert, 2004; Yoder et al., 2003). We measured morphological characters and performed Restriction site Associated DNA sequencing (RAD‐seq) to assess the effect of the River Niger on the distribution of genotypic and morphological diversity in *C. platycephalus*, and how well morphological and molecular data provide congruent information on the distribution of intraspecific variation.

## 2 MATERIALS AND METHODS

### 2.1 Study area and specimens

**Figure 2:**
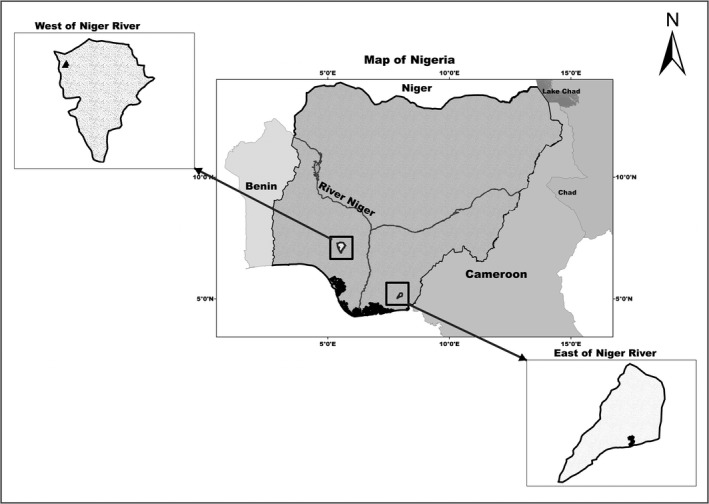
Map of Nigeria showing the origin of the populations used in this study. The black regions in the insets show the sampling locations

Specimens were collected from two eco regions in Nigeria (Figure 2): 16 from west of the River Niger, generally described as Guinea forest, savanna mosaic, and Nigerian lowland forest (N 07^°^ 14′ 09.307″, E 005^°^ 25′ 15.761″); and 17 from east of the River Niger (also known as southern Nigeria), generally described as Niger Delta Swamp Forest, Cross‐Niger Transition Forest, Cross‐Sanaga‐Bioko Coastal Forest, and Northwestern Congolian Lowland Forest (N 05^°^ 06′ 19.056″, E 008^°^ 00′ 03.749″) (Burgess et al., 2004). Roadside kills were collected from hunters outside of protected regions. No institutionally approved protocol was required for this research because tissues were collected from animals that were already dead, the species is not in danger of extinction, and they were obtained outside of protected regions. The sixteen individuals from the west were collected from hunters as carcasses within 12 hr after death and thus considered fresh kills, while the 17 specimens from the east were collected approximately 72 hr after death (due to distance of the collection site from the laboratory).

### 2.2 Morphological differentiation

We measured the following external morphological traits in each individual from the two sampling locations: total length, snout length, tail length, and limb length were each measured on the carcasses using a board and tape where appropriate, following the methodology of Kingdon et al. (2013). The mean value of each measurement was calculated for each population, and these means were compared between the two populations using two‐sample *t* tests. We did not consider body weight in the analysis because postmortem desiccation decreases weight, and our individuals were deceased for different lengths of time prior to measurement. We used the function ‘prcomp’ in the R statistical programming environment (R Core Team 2017) to perform a principal components analysis (PCA) on the morphological measurements. The variables were scaled to have unit variance and zero‐centered prior to analysis.

### 2.3 DNA samples

Liver, ear, and skin tissue samples were collected from the 33 individuals of *C. platycephalus*, 16 individuals from west of the River Niger, and 17 from east of the River Niger. Samples were preserved in 95% ethanol for a period of approximately 6 month before taking them to the Department of Biology and Biochemistry, University of Houston. Prior to DNA extraction, samples were preserved at −20°C. DNA was extracted from the tissue using a Masterpure DNA purification kit (Epicentre/Illumina), following the manufacturer's instructions. DNA quality and concentration was assessed with agarose electrophoresis and with a NanoDrop spectrophotometer (Promega, Inc.).

### 2.4 RAD‐seq library preparation

We used DNA from the 33 samples to construct RAD‐seq libraries following the instructions in a published protocol (Parchman et al., 2012), unless otherwise specified. Purified DNA was digested with the endonucleases EcoRI and MseI, barcoded adapters were ligated at restriction cut sites, and PCR primers were used to amplify the individual libraries. We did not use gel extraction to size‐select the libraries because yield was too low. Instead, the amplified PCR products were cleaned using AMPure XP beads (Agencourt) in the ratio 0.8–1 of AMPure bead to PCR product solution, which selected fragments of at least 250 bp. The length and concentration of libraries were assessed with a NanoDrop spectrophotometer and quantitative electrophoresis in a Bioanalyzer (Agilent, Inc.).

### 2.5 Sequencing and analysis

Sequencing was performed using a medium output, 75 cycle (base pair), single‐end run on an Illumina NextSeq500 machine at the University of Houston Seq‐N‐Edit Core, following the manufacturer's instructions. The reads were demultiplexed using the Illumina bcl2fastq software and assembled using the STACKS pipeline (Catchen, Hohenlohe, Bassham, Amores, & Cresko, 2013). Default parameters were used unless otherwise specified. In order to include sufficient loci for analysis, it was necessary to use liberal thresholds in the STACKS pipeline. This is likely because of DNA degradation in our older biological samples (Graham et al., 2015). Raw reads from sequencing were first passed through process_radtags in the Stacks pipeline, with the following options: discard reads with low‐quality scores (‐q), a score limit (‐s) of 5, truncating reads (‐t) to 65 bp, disabled checking if the restriction site is intact (–disable_rad_check), and barcode rescue (‐r) with up to three mismatches allowed (–barcode_dist_1 3). We excluded the verification of enzyme cut sites because most reads did not have perfect cut site matches. Next, ustacks was run to align sequences into matching stacks (loci) and detect single nucleotide polymorphisms (SNPs), with default parameters, including a minimum read depth (‐m) of 3 to retain reads in a stack. This was followed by cstacks to build a catalog, create consensus loci, and merge alleles, with three mismatches allowed between sample loci (‐n 3). We used sstacks to match alleles against the catalog, and rxstacks was used to correct the genotype and haplotype calls based on population‐wide analyses with the following options: up to 75% of loci in a population can be confounded relative to the catalog locus (–conf_lim 0.75) and prune out nonbiological haplotypes unlikely to occur in the population (–prune_haplo). We then reran cstacks and sstacks on the corrected calls. The populations program was next used to compute population‐based summary of statistics such as *F*_ST_ and *F*_IS_, with the following options: a locus may be present in a single population (‐p 1), 25% of individuals in a population were required to have a genotype for each locus (‐r 0.25), kernel‐smoothed calculations of population statistics was enabled (‐k), and SNP and haplotype‐based F statistics were calculated (–fstats). For output into downstream analyses (*e.g.,* STRUCTURE), we reran the populations program with the option to only write the first SNP from any RAD locus (–write_single_snp) because this reduces the inclusion of linked data in the downstream analyses. Finally, STRUCTURE and GENEPOP were used to determine the genetic structure of the populations and calculate population genetics metrics using the variant calls from Stacks (Pritchard, Stephens, & Donnelly, 2000; Raymond & Rousset, 1995; Rousset, 2008). The following STRUCTURE settings were used: 5,000 generation burn‐in period, 50,000 MCMC reps, the admixture model was activated under ancestry mode, and allele frequency was correlated among population.

## 3 RESULTS

### 3.1 Morphometric differentiation

**Figure 3:**
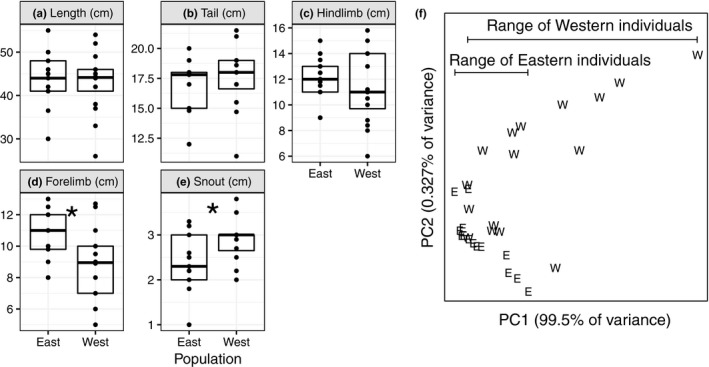
Distributions of (a) body length, (b) tail length, (c) hind limb length, (d) fore limb length, and (e) snout length in the two populations. Each point is an individual, and the boxes show the median (midline) and quartiles (top and bottom of box) of the distributions. Significant differences (*p* < 0.05 in Welch's *t* test) between the east and west population are indicated by an asterisk. (f) The loadings for each individual from east of the River Niger (“E”) and west of the River Niger (“W”) in the first two PCs are plotted. The ranges of individuals from each population along PC1 are also shown

We sampled *C. platycephalus* from east and west of the River Niger in Nigeria, and we measured five morphological traits in those individuals (Supporting Information Table S1). We compared the individual morphological measurements between the western and eastern populations (Figure 3a–e). There is not a significant difference in total length (*p* = 0.94), tail length (*p* = 0.23), or hind limb length (*p* = 0.43) between the populations. Fore limbs were larger in the east (*p* = 0.01), and snout length is longer in the west (*p* = 0.01). We additionally used a principal component (PC) analysis to group individuals based on the five length measurements. Individuals from west of the River Niger have more variation in the first PC (PC1, which explains >99% of the variance) than those from the eastern population (Figure 3f).

### 3.2 Genetic differentiation

**Figure 4:**
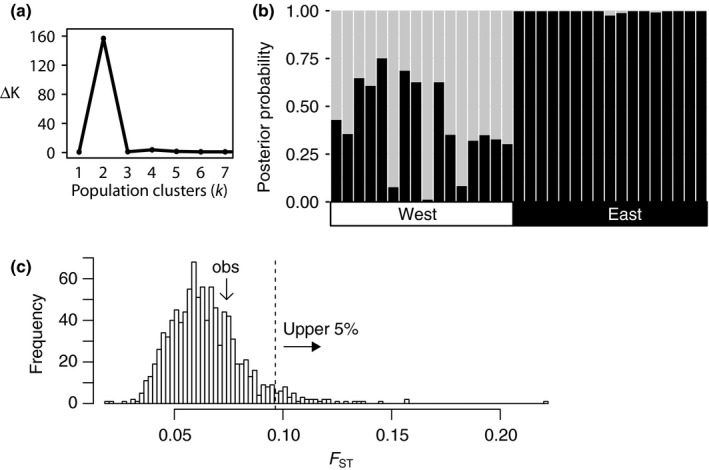
(a) Estimate of the true value of *k* (the number of clusters). The peak of Δ*K* at *k *=* *2 suggests that there are two population clusters. (b) Population clusters from STRUCTURE. Each column is an individual who is from either west of River Niger (left grouping) or east of River Niger (right grouping). The gray shading indicates the proportion of each individual's genotype that was assigned to a genotype cluster that is most common to the west of River Niger, and the black shading is the proportion of each genotype that was assigned to a cluster that predominates in the individuals from east of River Niger. (c) The estimate of *F*_ST_ between east and west populations is compared to randomized *F*_ST_ values. The arrow shows the observed value of *F*_ST_ between the populations from east and west of the River Niger. The distribution shows the frequency of *F*_ST_ estimates when the 33 individuals were randomly assigned to two populations with 17 and 16 individuals each. The dashed black line is the cut‐off separating the lower 95% randomized *F*_ST_ values from the upper 5%

We obtained 27,265,914 RAD‐seq reads in total from 33 libraries constructed from the individuals we sampled from west and east of the River Niger, of which 12,190,295 reads passed our quality filters. Our Stacks pipeline identified 77 loci in these data, which we used to assign individuals to populations using STRUCTURE (Pritchard et al., 2000). We estimated the true value of *k* (the number of clusters or source populations) by plotting Δ*K* (change in the log probability of data with respect to the number of clusters) against successive *k* values (Evanno, Regnaut, & Goudet, 2005). A peak of Δ*K* at *k *=* *2 suggests that there are two clusters within our sample (Figure 4a). We then used STRUCTURE to assign the genotypes of our 33 individuals to the two clusters. Individuals from east of the River Niger are almost completely assigned to a single cluster, but most individuals from the west of the River Niger have genotypes that are a mixture of the two clusters (Figure 4b).

Despite the genotypic differentiation between the western and eastern populations detected by STRUCTURE, the observed *F*_ST_ between populations is low (0.074). We calculated a null distribution of *F*_ST_ assuming no population subdivision by randomly assigning the 33 individuals to two populations over 1,000 iterations. The observed *F*_ST_ value does not fall in the upper 5% tail of the null distribution (Figure 4c), suggesting that there is not genetic differentiation between the western and eastern populations. This result conflicts with the inference from the STRUCTURE analysis, and it could be a result of the ability of STRUCTURE to identify genotype clusters even when F_ST_ values are low (Evanno et al., 2005; Pritchard et al. 2000).

We next examined the genetic variation within the populations from west and east of the River Niger. Observed homozygosity is higher in the population east of the River Niger, and the population from west of the River Niger has higher observed heterozygosity (Table 1). There is not a significant difference in expected homozygosity or expected heterozygosity between the populations (Table 1)*,* suggesting that the differences in observed homozygosity and heterozygosity are not only due to differences in overall genetic variation between the populations. There is also not a significant difference in the inbreeding coefficient (*F*_IS_) between the populations from west and east of the River Niger (Table 1).

**Table 1:** Measure of genetic variation in the populations

Diversity measure	West of Niger	East of Niger	*p*‐value
Mean observed homozygosity	0.779 ± 0.11	0.981 ± 0.06	0.0003
Mean expected heterozygosity	0.685 ± 0.14	0.897 ± 0.17	0.12
Mean observed heterozygosity	0.220 ± 0.11	0.019 ± 0.06	0.0003
Mean expected heterozygosity	0.279 ± 0.17	0.138 ± 0.18	0.11
Inbreeding coefficient	0.176 ± 0.22	0.410 ± 0.50	0.22

*Note*

The mean values are shown, along with the standard deviations. *p*‐Values are from a two‐sample *t* test.

## 4 DISCUSSION

We performed both morphological and genotypic analyses of flat‐headed cusimanse, *C. platycephalus*, from west and east of the River Niger in Nigeria (Figure 2). The morphological and molecular data both lead us to conclude that there is greater diversity in the western population, with the eastern population containing a subset of the phenotypic and genetic diversity found in the eastern population (Figures 3 and 4). In addition, there is elevated homozygosity (and reduced heterozygosity) in the eastern population (Table 1). These results demonstrate that combining genotypic and phenotypic diversity measures can provide congruent information about the distribution of intraspecific variation that could possibly be used to strengthen conservation recommendations.

Of the five morphological characters that we measured, only snout and fore limb lengths are significantly different between individuals sampled from west and east of the River Niger (Figure 3). The mean snout length is longer in the western population. The common cusimanse (*C. obscurus*) has a longer snout length than *C. platycephalus*, and *C. obscurus* also occurs west of the River Niger (Sonet et al., 2014). In addition, molecular data do not completely resolve the monophyly of *C. platycephalus* (Sonet et al., 2014). One possible explanation for the longer snout length in the western population of *C. platycephalus* is archaic hybridization with *C. obscurus*. Both species are capable of living in similar habitats (Angelici & Di Vittorio, 2013), but their ranges are currently separated by the Dahomey Gap, a large, dry, open area that extends through Benin, Togo, and eastern Ghana (Angelici & Di Vittorio, 2013; Sonet et al., 2014). The extent of the Dahomey gap has fluctuated over the past 150,000 years (Dupont & Weinelt, 1996; Maley, 2001; Salzmann & Hoelzmann, 2005), suggesting that the ranges of *C. obscurus* and *C. platycephalus* may have overlapped during a period when they could have hybridized. Archaic hybridization with *C. obscurus* could have introduced genetic variation into the western *C. platycephalus* population, which would explain the elevated molecular and morphological diversity west of the River Niger.

There are at least four alternative explanations for why *C. platycephalus* from east of the River Niger have less genotypic and morphological variation than west of the River Niger. First, there could be more migration from the east to the west of the river, than vice versa, increasing genetic diversity in the western population. Second, the reduced genetic diversity in the east could be the result of a founder event sampling only a subset of the genetic diversity found in the west. Third, both populations could have arisen from a common ancestral population, with the eastern population losing more of the genetic diversity than was retained in the west. Finally, a recent ecological disturbance may have reduced the size of the eastern population. This final hypothesis is supported by the observation that there are fewer tree species east of the River Niger, and trees in the east have lower average diameters than those to the west of the river (Oguntuase, 2017). This is consistent with more ecological disturbance east of the river, although there could be other explanations for the forest diversity (Connell, 1978; Niklas, Midgley, & Rand, 2003). Additional morphological and population genetic data from *C. platycephalus* and *C. obscurus* are needed to test these five hypotheses.

An important caveat to our conclusions is that we only measured morphological and molecular variation in one sample from each side of the River Niger. Moreover, the individuals were obtained from hunters, and the exact location of capture is unknown. Additional sampling from multiple specific locations from each side of the river would allow for comparisons of variation among western populations, among eastern populations, and across the species range. These data and analyses would allow for additional tests of decreased variation in the eastern population, and also whether differentiation between western and eastern populations exceeds variation on each side of the River Niger.

If additional analyses continue to support the observation that there is increased genetic diversity west of the River Niger relative to east of the river, it would suggest that the River Niger may be an important factor to consider in conservation of endangered species. For example, if lower genetic diversity in the eastern population were a result of a recent reduction in population size because of habitat disturbance, a natural or man‐made event that could have affected *C. platycephalus* could have also affected other species that are under more imminent threat of extinction. In addition, if the Dahomey Gap has not always served as a barrier between species in western sub‐Saharan Africa, surveys of genetic variation in this region should consider the effect of ancient gene flow across the Dahomey Gap on the distribution of extant variation. In conclusion, our results demonstrate that concurrent analysis of both morphological and genotypic diversity can provide a congruent view of the distribution of intraspecific variation. Future work is necessary to further evaluate the role of the River Niger in the distribution of genetic variation in the animals of Nigeria.

## CONFLICT OF INTEREST

None declared.

## AUTHOR CONTRIBUTIONS

BG Oguntuase, BG Ogunjemite, and RP Meisel designed the research; BG Oguntuase performed the research; BG Oguntuase and RP Meisel analyzed the data and wrote the paper.

## DATA ACCESSIBILITY

The RAD‐seq data generated in this study are available in the NCBI Sequence Read Archive under accessions SRR5926961–SRR5926993 (BioProject PRJNA397925).
