# Supplementary material for: Morphometric and genetic differentiation among populations of flat‐headed cusimanse (Crossarchus platycephalus) in Nigeria
Source: Ecol Evol. 2018 Jun 25;8(14):7228–35. doi: 10.1002/ece3.4262 (PMC6065274; doi:10.1002/ece3.4262)
Supplement: Supplementary file 1 [file ECE3-8-7228-s001.docx]

**Supplementary Table S1: Morphometrics of specimens collected from the two locations in Nigeria**

| **Population** | **Total length (cm)** | **Tail length (cm)** | **Hindlimb (cm)** | **Forelimb (cm)** | **Snout length (cm)** |
| --- | --- | --- | --- | --- | --- |
| West of Niger | 46.0 | 19.0 | 11.0 | 7.0 | 3.0 |
| West of Niger | 45.0 | 17.0 | 14.0 | 10.0 | 3.5 |
| West of Niger | 44.0 | 21.0 | 14.0 | 9.5 | 3.0 |
| West of Niger | 44.0 | 18.0 | 11.0 | 6.0 | 3.0 |
| West of Niger | 42.0 | 17.0 | 8.0 | 5.0 | 3.0 |
| West of Niger | 26.0 | 11.0 | 6.0 | 6.0 | 2.0 |
| West of Niger | 44.3 | 19.0 | 13.0 | 10.0 | 3.0 |
| West of Niger | 52.0 | 18.0 | 14.0 | 11.0 | 3.0 |
| West of Niger | 42.0 | 18.0 | 10.0 | 8.0 | 2.7 |
| West of Niger | 46.0 | 19.0 | 8.8 | 7.0 | 2.9 |
| West of Niger | 45.0 | 18.6 | 11.2 | 9.0 | 3.0 |
| West of Niger | 38.0 | 15.5 | 8.4 | 7.0 | 2.2 |
| West of Niger | 54.0 | 18.0 | 15.8 | 12.7 | 3.8 |
| West of Niger | 49.0 | 21.5 | 15.0 | 12.5 | 3.5 |
| West of Niger | 37.0 | 15.5 | 11.0 | 10.0 | 2.5 |
| West of Niger | 33.0 | 14.7 | 10.5 | 8.9 | 2.0 |
| East of Niger | 45.0 | 18.0 | 12.5 | 12.0 | 2.5 |
| East of Niger | 44.0 | 18.0 | 13.0 | 11.0 | 2.6 |
| East of Niger | 30.0 | 12.0 | 9.0 | 8.0 | 1.0 |
| East of Niger | 30.0 | 12.0 | 9.0 | 8.0 | 1.0 |
| East of Niger | 30.0 | 12.0 | 9.0 | 8.0 | 1.0 |
| East of Niger | 41.0 | 15.0 | 11.0 | 9.8 | 2.2 |
| East of Niger | 50.0 | 20.0 | 14.0 | 13.0 | 3.2 |
| East of Niger | 46.0 | 19.0 | 14.0 | 12.0 | 3.2 |
| East of Niger | 48.0 | 18.0 | 12.0 | 10.0 | 3.2 |
| East of Niger | 41.0 | 15.0 | 12.5 | 11.0 | 2.2 |
| East of Niger | 50.0 | 19.0 | 13.5 | 12.5 | 3.0 |
| East of Niger | 50.0 | 18.0 | 13.0 | 12.0 | 2.5 |
| East of Niger | 48.0 | 17.8 | 12.0 | 11.0 | 2.0 |
| East of Niger | 36.5 | 14.8 | 11.8 | 10.0 | 1.8 |
| East of Niger | 46.0 | 19.0 | 11.0 | 7.0 | 3.0 |
| East of Niger | 45.0 | 17.0 | 14.0 | 10.0 | 3.5 |
| East of Niger | 44.0 | 21.0 | 14.0 | 9.5 | 3.0 |

**Supplementary Table S2: Loadings of principal components (PCs) from morphological analysis**

| **Population** | **PC1** | **PC2** | **PC3** | **PC4** | **PC5** |
| --- | --- | --- | --- | --- | --- |
| West of Niger | -0.17368 | 0.28655 | -0.01517 | 0.17133 | -0.86360 |
| West of Niger | -0.17418 | -0.05300 | -0.14156 | 0.40810 | 0.21229 |
| West of Niger | -0.17356 | 0.15803 | 0.43723 | 0.30582 | 0.00065 |
| West of Niger | -0.17341 | 0.30691 | -0.03683 | 0.36948 | 0.24162 |
| West of Niger | -0.17270 | 0.42638 | -0.15648 | -0.00294 | 0.10438 |
| West of Niger | -0.17403 | 0.14730 | -0.01157 | -0.43214 | 0.03821 |
| West of Niger | -0.17437 | 0.06090 | 0.15170 | 0.12524 | 0.03676 |
| West of Niger | -0.17419 | -0.06834 | -0.27166 | 0.17347 | -0.08529 |
| West of Niger | -0.17398 | 0.22549 | 0.07567 | -0.11357 | 0.03792 |
| West of Niger | -0.17327 | 0.34820 | -0.07087 | -0.22150 | 0.19736 |
| West of Niger | -0.17426 | 0.15738 | 0.00402 | -0.05524 | 0.07221 |
| West of Niger | -0.17404 | 0.20808 | -0.02645 | -0.17441 | 0.00374 |
| West of Niger | -0.17373 | -0.17271 | -0.36462 | 0.24771 | 0.05226 |
| West of Niger | -0.17436 | -0.00696 | 0.21466 | 0.01969 | 0.00737 |
| West of Niger | -0.17436 | -0.08668 | 0.11738 | -0.11987 | 0.04699 |
| West of Niger | -0.17414 | -0.07068 | 0.32266 | 0.01189 | -0.00043 |
| East of Niger | -0.17446 | 0.04163 | 0.05317 | 0.07822 | 0.03914 |
| East of Niger | -0.17433 | -0.10309 | -0.14689 | -0.05414 | -0.07661 |
| East of Niger | -0.17409 | -0.13663 | -0.27793 | 0.04254 | 0.04109 |
| East of Niger | -0.17430 | -0.11156 | 0.04305 | -0.22575 | -0.05942 |
| East of Niger | -0.17442 | -0.06755 | 0.09644 | 0.02000 | 0.10330 |
| East of Niger | -0.17407 | -0.18651 | 0.17761 | -0.03568 | -0.01354 |
| East of Niger | -0.17407 | -0.18651 | 0.17761 | -0.03568 | -0.01354 |
| East of Niger | -0.17407 | -0.18651 | 0.17761 | -0.03568 | -0.01354 |
| East of Niger | -0.17433 | -0.10309 | -0.14689 | -0.05414 | -0.07661 |
| East of Niger | -0.17440 | -0.08148 | 0.00515 | -0.16203 | 0.06586 |
| East of Niger | -0.17441 | -0.08201 | 0.08460 | 0.01753 | 0.04840 |
| East of Niger | -0.17436 | 0.04982 | -0.19161 | -0.02479 | 0.12285 |
| East of Niger | -0.17393 | -0.23899 | -0.08913 | 0.06610 | -0.07457 |
| East of Niger | -0.17436 | -0.09161 | -0.09823 | -0.14057 | -0.04779 |
| East of Niger | -0.17427 | -0.11251 | -0.17145 | -0.11952 | -0.13166 |
| East of Niger | -0.17440 | -0.06027 | -0.09241 | -0.15404 | -0.04221 |
| East of Niger | -0.17401 | -0.20206 | 0.17012 | 0.10746 | 0.02678 |
